# A case report of an open aortic valve replacement followed by open adrenalectomy in a patient with symptomatic pheochromocytoma and critical aortic stenosis

**DOI:** 10.1186/s13019-021-01665-x

**Published:** 2021-09-28

**Authors:** Igor Feinstein, Tiffany Lee, Sameer Khan, Lindsay Raleigh, Frederick Mihm

**Affiliations:** 1 Department of Anesthesiology, Perioperative and Pain Medicine, Stanford University Medical Center, Rm H3580, 300 Pasteur Drive, Stanford, CA 94305 USA; 2 The Permanente Medical Group, San Francisco Medical Center, 2238 Geary Blvd. 8th Floor, San Francisco, CA 94115 USA; 3 Divisions of Adult Cardiothoracic Anesthesiology and Critical Care Medicine, Department of Anesthesiology, University of Southern California (Keck + LAC), 1450 San Pablo Street, Suite 3600, Los Angeles, CA 90033 USA

**Keywords:** Aortic stenosis, Pheochromocytoma, Aortic valve replacement, Vasoplegic shock, Case report

## Abstract

**Background:**

Pheochromocytoma is a rare medical condition caused by catecholamine-secreting tumor cells. Operative resection can be associated with significant hemodynamic fluctuations due to the nature of the tumor, as well as associated post-resection vasoplegia. To allow for cardiovascular recovery before surgery, patients require pre-operative alpha-adrenergic blockade, which would be limited in the setting of co-existent severe aortic stenosis. In this report, we describe a patient with severe aortic stenosis and symptomatic pheochromocytoma.

**Case presentation:**

A 51-year-old man with severe aortic stenosis (valve area 0.8 cm^2^) was found to have a highly active 4 × 4 cm left adrenal pheochromocytoma. Alpha-adrenergic blockade for his pheochromocytoma was limited by syncope in the setting of his aortic stenosis. Open aortic valve replacement (AVR) was performed, followed by adrenalectomy the next day. The perioperative course for each surgical procedure was hemodynamically volatile, exacerbated by severe alcohol withdrawal. During the adrenalectomy, cardiogenic and vasoplegic shock developed immediately after securing the vascular supply to his tumor. This shock was refractory to vasopressin and methylene blue, but responded well to angiotensin II and epinephrine. After both surgeries were completed, his course was further complicated by severe ICU psychosis, ileus, fungal bacteremia, pneumonia/hypoxic respiratory failure and atrial fibrillation. He ultimately recovered and was discharged from the hospital after 38 days.

**Conclusion:**

To our knowledge, this is the first report of surgical AVR and pheochromocytoma resection in a patient with critical aortic stenosis. The appropriate order and timing of surgeries when both these conditions co-exist remains controversial.

## Background

Pheochromocytoma is a rare medical condition caused by the secretion of catecholamines by chromaffin tumor cells, usually in the adrenal medulla. Current guidelines recommend treatment of symptomatic pheochromocytomas with α-adrenergic blockade to blunt the effects of elevated catecholamine output and allow cardiovascular recovery prior to surgical resection [1, 2]. However, adequate α-blockade can be challenging in the setting of severe aortic stenosis, as the hemodynamic goals of these two pathologies are conflicting. Surgical treatment of patients with severe aortic stenosis and pheochromocytoma remains a high-risk procedure with fatal complications [3, 4].

To our knowledge, this is the first reported case of combined critical aortic stenosis and pheochromocytoma where a two-day staged procedure of open aortic valve replacement (AVR) and subsequent adrenalectomy was performed. Written consent was obtained from the patient.

## Case presentation

A 51-year-old man with a history of a heart murmur since childhood presented with periodic palpitations, headaches, flushing, nausea and vomiting. Transthoracic echocardiography (TTE) revealed severe aortic stenosis with an aortic valve area of 0.8 cm^2^ and a mean gradient of 48 mmHg, as well as mild-moderate aortic regurgitation. During further workup, the patient was also found to have a functional 4 × 4 cm left adrenal pheochromocytoma with elevated plasma metanephrine and normetanephrine of 4.88 (nl 0–0.49) and 4.44 (nl 0–0.89) nmol/L respectively. Outpatient management of his pheochromocytoma included terazosin 2 mg twice daily, bisoprolol 10 mg daily, losartan 50 mg twice daily, and amlodipine 2.5 mg twice daily. Treatment with α-blockade was limited due to his severe aortic stenosis, as the patient experienced syncopal episodes with increased doses of terazosin. A staged surgical approach was planned: surgical AVR followed by adrenalectomy the next day.

The patient was brought to the operating room where femoral arterial and venous access was obtained under sedation using dexmedetomidine and midazolam. Anesthesia was induced with fentanyl, midazolam, propofol, and rocuronium. Intubation was performed with 4% lidocaine topicalization and video laryngoscopy to minimize sympathetic stimulation. Intraoperative analgesia was augmented with a high dose sufentanil infusion and incremental boluses as needed. A clevidipine infusion and incremental clevidipine boluses were used to control very labile mean arterial pressures (MAP) up to 150 mmHg. Cannulation and initiation of cardiopulmonary bypass (CPB) were otherwise uneventful.

**Fig. 1:**
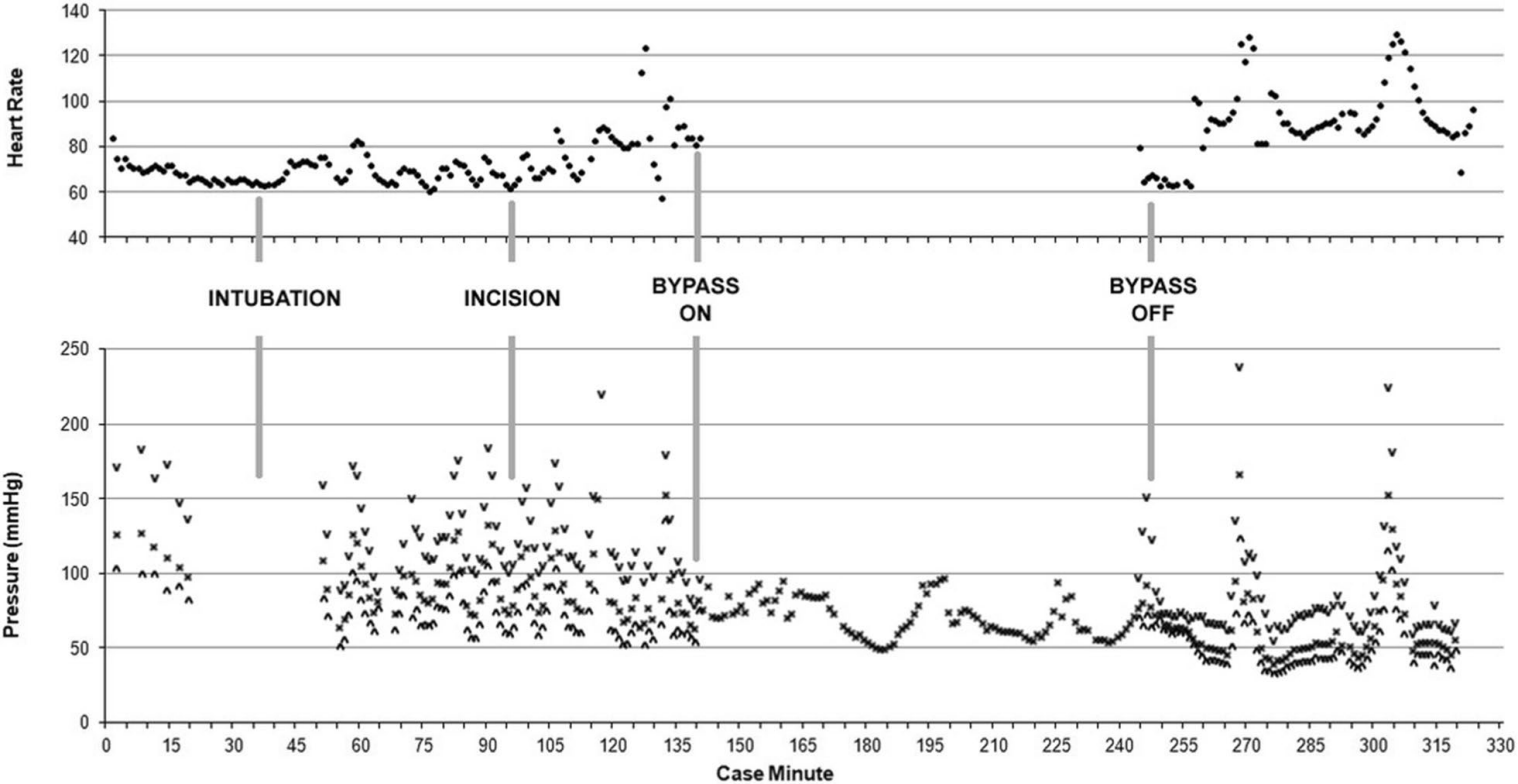
AVR intraoperative hemodynamics (Note: some BP data missing between minute 16–50 min)

While on CPB, significant hypertension required clevidipine and nitroglycerin (NTG) infusions. Following placement of a 23 mm St. Jude mechanical valve in the aortic position (mean gradient of 10 mmHg), the patient was weaned from CPB with normal biventricular function. The immediate post-CPB period was marked by profound hemodynamic lability (MAP < 50 to > 150 mmHg) (Fig. 1). Severe hypertension required large boluses of NTG and clevidipine; and severe hypotension was treated with vasopressin. Of note, the patient was hyperglycemic throughout the case with glucose levels exceeding 500 mg/dL, requiring a high dose insulin infusion.

After transfer to the intensive care unit (ICU), in an attempt to control hemodynamics and blunt any sympathetic discharges, he was deeply sedated with high doses of midazolam (8 mg/hr), hydromorphone (8 mg/hr) and dexmedetomidine (1.7 mcg/kg/min). He was also paralyzed with a cisatracurium infusion to assist with ventilator synchrony. Despite these interventions, dramatic blood pressure swings continued, reaching MAP of 150–175 mmHg and requiring maximum doses of clevidipine, sodium nitroprusside, esmolol and fenoldopam, followed by extreme hypotension with MAPs 35–40 mmHg. These cyclical events recurred more than a dozen times despite continuous bedside physician attention and best attempts at drug titration. The patient also developed high fevers to 39.4 °C and severe hyperglycemia requiring a high dose insulin infusion with additional boluses of insulin for glucose levels exceeding 300 mg/dl. During this time, the patient's wife admitted that the patient was drinking alcohol heavily up until the day before surgery. This raised our concern that acute alcohol withdrawal was contributing to his labile state.

The morning after his AVR, the patient remained very unstable with extremely labile blood pressures and began to develop runs of non-sustained ventricular tachycardia for which he was started on amiodarone. Because of the high likelihood of significant morbidity/mortality if the pheochromocytoma was not removed, the patient was taken urgently to the operating room for open adrenalectomy.

**Fig. 2:**
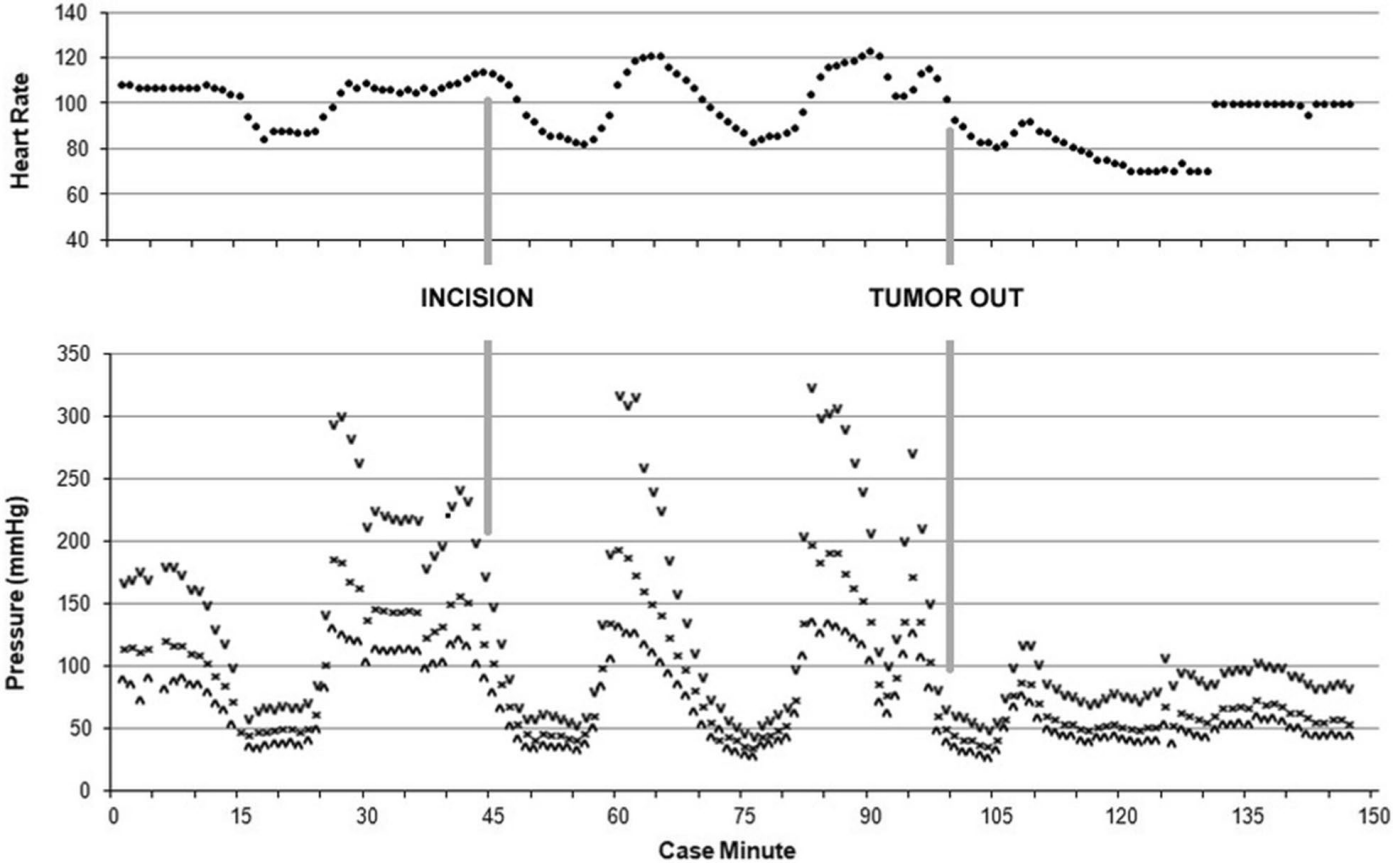
Pheochromocytoma resection intraoperative hemodynamics

Deep sedation and analgesia were maintained with the addition of low dose sevoflurane and a high dose remifentanil infusion. Intra-operatively, the patient continued to have labile hemodynamics unrelated to surgical stimulation, with MAPs ranging from 40 to 175 mmHg (Fig. 2). Escalating bolus doses of nitroprusside and nitroglycerin were given with minimal effect during these acute hypertensive episodes. During periods of hypotension, vasopressin boluses were used, also with minimal effect. It was noted that during episodes of extreme hypertension, the patient exhibited signs of acute right ventricular (RV) failure manifested by acute rises in central venous pressure (CVP) up to 30 mmHg and hypoxemia as evidenced by reduced arterial oxygen saturation (S_a_O_2_) to 85–90%, which resolved with decreased blood pressure.

After the pheochromocytoma was isolated from its vascular supply, immediate cardiogenic and vasoplegic shock developed with MAP ranging from 35–45 mmHg and cardiac output (CO) of 3.4 L/min. An epinephrine infusion was initiated to support inotropy and vascular tone. Sedation was appropriately decreased. Vasopressin boluses and a 1 mg/kg dose of methylene blue were administered with marginal effect. Subsequently, an infusion of recombinant angiotensin II was started and rapidly up titrated to a maximum dose (80 ng/kg/min) with improvement of MAPs to 50–60 mmHg. Given the native heart rate of 65 bpm was inadequate to support cardiac output and MAP, epicardial pacing was initiated in DDD mode at 100 bpm. This intervention increased the MAP to greater than 60 mmHg and CO to 4.5 L/min (Fig. 2). Shortly after tumor removal, glucose levels dropped precipitously, so the insulin drip was stopped and dextrose was administered.

The patient was transported back to the ICU, where his postoperative course was complicated by continued cardiogenic and vasoplegic shock, RV failure, ventricular tachycardia and hypoxic respiratory failure with pulmonary edema. He continued on amiodarone, epinephrine and angiotensin II infusions. A milrinone infusion and inhaled epoprostenol was added for RV support with significant improvement in both oxygenation and CO (> 6 L/min). He remained sedated with dexmedetomidine, hydromorphone and midazolam. The patient was weaned off angiotensin II six hours postoperatively, followed by a deep sedation taper the following day.

Postoperative recovery was hindered by severe agitation and delirium with hallucinations, but on postoperative day (POD) 6 he was extubated and weaned off all inotropic and vasopressor support. His subsequent hospital course was marked by fungal infection (workup negative for endocarditis), methicillin-resistant Staphylococcus aureus (MRSA) pneumonia, atrial fibrillation, and respiratory failure requiring a period of intubation. His delirium finally resolved six weeks after surgery, and he was discharged home on POD 38.

## Discussion:

There have been numerous reports of cardiovascular crises associated with cardiac surgery in the presence of undiagnosed pheochromocytoma [5, 6]. Successful resection of pheochromocytomas in the setting of coronary artery bypass grafting (CABG) involving cardiopulmonary bypass (CPB) has been reported as both staged (CABG followed by pheochromocytoma resection and pheochromocytoma resection followed by CABG), as well as simultaneous CABG-pheochromocytoma resection procedures [7–9]. Severe aortic stenosis in combination with pheochromocytoma is a rare and high-risk situation and has also been associated with mortality in cases of undiagnosed pheochromocytomas [3, 4]. While resection of a symptomatic pheochromocytoma is strongly recommended and life-saving, valve replacement in the setting of symptomatic aortic valve stenosis is also necessary [10, 11]. Options for AVR include surgical or transcatheter aortic valve replacement (TAVR), or temporizing with balloon dilation. Saran et al. reported a successful resection of a pheochromocytoma in a patient with a pre-surgical aortic valve area of 0.9 cm^2^ and a mean gradient of 55 mmHg. However, since the post-pheochromocytoma resection aortic valve area was measured as 0.95 cm^2^ with a reduced mean gradient of 37 mmHg, they did not perform an AVR [12]. Henderson et al.presented a case of medical control of a pheochromocytoma followed by a TAVR in a 81 year old patient with an aortic valve area of 1.1 cm^2^ and a mean gradient of 57 mmHg. However, they did not surgically resect the pheochromocytoma and the patient remained medically managed [13].

In our case, a multidisciplinary meeting with anesthesiology, cardiology, cardiac surgery, and surgical oncology was held to discuss treatment options for critical aortic stenosis and pheochromocytoma. In selecting an appropriate treatment option, it was necessary to balance the desire for a durable repair for the patient’s aortic valve pathology while being cognizant of the serious and potentially lethal cardiovascular morbidity that can occur during uncontrolled catecholamine release from the pheochromocytoma. Complications that have been previously described include acute myocarditis, cardiac failure, Takotsubo/reverse Takotsubo cardiomyopathy, arrhythmias and aortic dissection [14–17]. Furthermore, use of cardiopulmonary bypass and systemic anticoagulation in the setting of a pheochromcytoma added unique considerations, including increased catecholamine levels after establishing bypass, potential hemorrhage in the tumor with retroperitoneal bleeding and severe hypertension and tachycardia after separation from cardiopulmonary bypass [18, 19]. To allow cardiac recovery and reduce intra-operative risks, guidelines recommend that patients with pheochromocytoma should receive effective pre-operative α-blockade for at least 1–2 weeks prior to tumor removal, however this was not possible for our patient because of his concomitant aortic stenosis [20–22].

Due to the patient’s elevated cardiovascular risk from the poorly optimized pheochromocytoma, minimally invasive approaches were considered first. Initially, the idea of pursuing a TAVR followed by pheochromocytoma resection was discussed. However, it was felt that given the patient’s young age and presence of concomitant aortic regurgitation, TAVR would not achieve a durable and reliable repair. There was also concern that if the patient experienced a blood pressure spike during valve deployment, it would place him at high risk of annular rupture or valve malposition, which would necessitate emergent surgical intervention. Additionally, TAVR would require immediate initiation of antiplatelet therapy which would cause challenges in planning for pheochromocytoma resection. Tumor embolization followed by AVR was also considered as an approach to control the activity of the catecholamine-secreting tumor prior to AVR. However, clinical experience suggests that embolization is an uncontrollable procedure, especially if the entire tumor infarcts. Significant swings in blood pressure, severe hypotension, asystole and death have been reported [23].

Combined surgical procedures were also considered. With regard to a combined procedure with pheochromocytoma resection followed by immediate AVR, the main concern was the potential for severe vasoplegic and cardiogenic shock that could occur after the pheochromocytoma was resected. Given that the patient was unable to tolerate even modest alpha-adrenergic blockade and experienced syncope with escalation of pre-operative medical therapy, the profound drop in circulating catecholamines after tumor removal would have placed the patient at risk of multiorgan malperfusion, even if supported by extracorporeal circulation. Combined with the expected post cross-clamp myocardial stunning and post-bypass vasoplegia, this approach was felt to be very risky. These concerns are supported by a report of a combined CABG/pheochromocytoma removal which resulted in refractory shock ultimately leading to the patient’s demise [8].

On the other hand, considering a combined procedure with the AVR performed first, we reasoned that there would be an unacceptably high risk of bleeding during the pheochromocytoma resection, given the anticoagulation requirements during CPB for an open AVR and subsequent post-bypass coagulopathy. Additionally, there was still a concern about post-bypass myocardial stunning and vasoplegia contributing to the expected deleterious vasoplegic effects of tumor removal.

Ultimately, the consensus decision was to perform an open aortic valve replacement first, followed by left adrenal resection the following morning. This staged approach would permit recovery time from post aortic cross-clamp ventricular dysfunction and post-CPB vasoplegia. As a mechanical valve was deemed to be the most appropriate prosthesis given the patient’s young age, this approach also considered the need for the initiation of long-term anticoagulation shortly after valve replacement. While it was understood that blood pressure control might not be ideal during the AVR, it was felt that this approach would provide the most durable repair while avoiding potentially catastrophic vasoplegia, if the pheochromocytoma was addressed first.

Our patient’s course was tumultuous and unique. He was heavily sedated since even minor stimulation can provoke robust hemodynamic responses in patients with active pheochromocytomas. His agitation and delirium required complete muscle relaxation in the perioperative period in order to facilitate ventilator synchrony and lung protective ventilation. Hemodynamic and echocardiographic monitoring was required to maintain euvolemia and manage his complicated vasoactive drug therapy. He required antiarrhythmic agents for life-threatening ventricular tachycardia. His glucose levels were incredibly difficult to manage with an insulin infusion until the pheochromocytoma was resected and then required aggressive glucose supplementation. He required cooling maneuvers for his hypermetabolic state in order to reduce oxygen demand. While it was not possible to predict the degree to which his blood pressure would skyrocket and plummet intra-operatively and during his ICU course post AVR, this instability was certainly complicated by his unexpected and profound alcohol withdrawal. If alcohol withdrawal had not occurred, it remains unclear to what degree “recovery” from open heart surgery could have been accomplished overnight to allow for a less eventful pheochromocytoma resection the following day.

Following pheochromocytoma tumor resection, epinephrine and vasopressin are useful therapies for hypotension. In this case, the severe vasodilation and hypotension following pheochromocytoma removal was unresponsive to continuous infusions and boluses of epinephrine and vasopressin. Blood pressure also failed to respond to methylene blue but did respond to an infusion of angiotensin II. Our patient also required increased chronotropy via cardiac pacing to support his cardiac output. We used vasopressin [24] and methylene blue [25] because both have been successfully used in treating vasoplegia after pheochromocytoma resection. The triple combination of catecholamines, vasopressin and angiotensin II has been shown in septic vasoplegic shock to rapidly improve mean arterial pressure while minimizing the potential for toxicity from monotherapy [26, 27]. Our patient responded to this approach. Recently, angiotensin II has also been used successfully to treat vasoplegic shock in a pheochromocytoma patient unresponsive to vasopressin [28]. Our experience with this patient also supports the use of angiotensin II to augment blood pressure recovery in pheochromocytoma patients.

Due to the unpredictable hemodynamic effects of both procedures, consideration was also given to the use of extracorporeal circulatory life support (ECLS). However, the hemodynamic disturbances of our patient were ultimately not conducive for initiation of ECLS. The oscillating hypotension and severe hypertension seen after AVR would have made management of extracorporeal support challenging and, in the absence of myocardial dysfunction, would have had questionable benefit in improving organ perfusion. After pheochromocytoma resection, the patient did develop evidence of myocardial dysfunction but we were able to achieve stability with a combination of inotropic and vasopressor infusions and a higher pacing rate. While the benefit of ECLS in pure vasoplegic states has not been definitively established, it would have been a reasonable option in refractory combined cardiogenic and vasoplegic shock [29].

## Conclusions

Critical aortic stenosis in the setting of a pheochromocytoma remains a high-risk situation requiring a multidisciplinary discussion to plan the safest treatment option for each patient. Our experience provides a successful example of a staged procedure involving an open aortic valve replacement followed by an open pheochromocytoma resection the following day. While hemodynamic lability was expected, our patient experienced a multi-system exaggerated response to the staged procedure, requiring vigilance, prompt action and efficient communication between the anesthesiologist/critical care specialist and multidisciplinary colleagues. This case was impressively confounded by severe alcohol withdrawal. Additional experience will be needed to determine which anesthetic/surgical approach is optimal for these high-risk critically ill patients.

## Data Availability

All data generated or analysed during this study are included in this published article.

## Abbreviations

AVR: Aortic valve replacement
TTE: Transthoracic echocardiography
MAP: Mean arterial pressure
CPB: Cardiopulmonary bypass
NTG: Nitroglycerin
ICU: Intensive care unit
RV: Right ventricular
CVP: Central venous pressure
SaO2: Arterial oxygen saturation
CO: Cardiac output
POD: Postoperative day
MRSA: Methicillin-resistant Staphylocccus aureus
CABG: Coronary artery bypass grafting
TAVR: Transcatheter aortic valve replacement
